# Green-Synthesized Silver and Selenium Nanoparticles Using Berberine: A Comparative Assessment of In Vitro Anticancer Potential on Human Hepatocellular Carcinoma Cell Line (HepG2)

**DOI:** 10.3390/cells13030287

**Published:** 2024-02-05

**Authors:** Azza M. Khaled, Mohamed S. Othman, Sofian T. Obeidat, Ghada M. Aleid, Shimaa M. Aboelnaga, Alaa Fehaid, Heba M. R. Hathout, Ashraf A. Bakkar, Ahmed E. Abdel Moneim, Islam M. El-Garawani, Dalia S. Morsi

**Affiliations:** 1 Biochemistry Department, College of Medicine, University of Ha’il, Hail P.O. Box 2440, Saudi Arabia; a.khaled@uoh.edu.sa (A.M.K.); biostar55@yahoo.com (M.S.O.); g.aleid@uoh.edu.sa (G.M.A.); 2 Basic Sciences Department, Deanship of Preparatory Year, University of Ha’il, Hail P.O. Box 2440, Saudi Arabia; s.obeidat@uoh.edu.sa (S.T.O.); shimaasmart@yahoo.com (S.M.A.); 3 Forensic Medicine and Toxicology Department, Faculty of Veterinary Medicine, Mansoura University, El Mansoura 35516, Egypt; alaafehaid@mans.edu.eg; 4 Natural Resources Department, Faculty of African Postgraduate Studies, Cairo University, Giza 12613, Egypt; hathoutheba@cu.edu.eg; 5 Faculty of Biotechnology, October University for Modern Science and Arts (MSA), Giza 12566, Egypt; abakkar@msa.edu.eg; 6 Zoology and Entomology Department, Faculty of Science, Helwan University, Ain Helwan, Cairo 11795, Egypt; 7 Zoology Department, Faculty of Science, Menoufia University, Shibin El Kom 32511, Egypt; dr.garawani@science.menofia.edu.eg (I.M.E.-G.); dalia.sami@science.menofia.edu.eg (D.S.M.)

**Keywords:** berberine, selenium nanoparticles, silver nanoparticles, cyclin D, HepG2, apoptosis

## Abstract

A well-known natural ingredient found in several medicinal plants, berberine (Ber), has been shown to have anticancer properties against a range of malignancies. The limited solubility and bioavailability of berberine can be addressed using Ber-loaded nanoparticles. In this study, we compared the in vitro cytotoxic effects of both Ber-loaded silver nanoparticles (Ber-AgNPs) and Ber-loaded selenium nanoparticles (Ber-SeNPs) in the human liver cancer cell line (HepG2) and mouse normal liver cells (BNL). The IC_50_ values in HepG2 for berberine, Ber-AgNPs, Ber-SeNPs, and cisplatin were 26.69, 1.16, 0.04, and 0.33 µg/mL, respectively. Our results show that Ber and its Ag and Se nanoparticles exerted a good antitumor effect against HepG2 cells by inducing apoptosis via upregulating p53, Bax, cytosolic cytochrome C levels, and caspase-3 activity, and the down-regulation of Bcl-2 levels. Similarly, incubation with Ber and both Ber-NPs (Ag and Se) led to a significant dose-dependent elevation in inflammatory markers’ (TNF-α, NF-κB, and COX-2) levels compared to the control group. In addition, it led to the arrest of the G1 cell cycle by depleting the expression of *cyclin D1* and *CDK-2* mRNA. Furthermore, Ber and both Ber-NPs (Ag and Se) caused a significant dose-dependent increase in LDH activity in HepG2 cells. Furthermore, our findings offer evidence that Ber and its nanoparticles intensified oxidative stress in HepG2 cells. Furthermore, the migration rate of cells subjected to berberine and its nanoforms was notably decreased compared to that of control cells. It can be inferred that Ber nanoparticles exhibited superior anticancer efficacy against HepG2 compared to unprocessed Ber, perhaps due to their improved solubility and bioavailability. Furthermore, Ber-SeNPs exhibited greater efficacy than Ber-AgNPs, possibly as a result of the inherent anticancer characteristics of selenium.

## 1. Introduction

On a worldwide basis, cancer poses a substantial social, economic, and public health risk. The most common cancers around the world are those of the liver, lung, colon, and stomach, and liver cancer ranks as the third most common in terms of global morbidity and mortality [1]. Berberine (Ber) is a small isoquinoline alkaloid present mainly in the stem and roots of various herbs within the *Berberis* genus [2]. It displays a variety of biological activities, such as anti-inflammatory [3], anti-diabetic [4], anti-hyperlipidemic [5], cardioprotective, memory-enhancing, and anti-depressant effects [6,7,8]. Previous research has suggested that Ber has strong potential as an anticancer agent due to its notable antitumor effects [9,10,11]. The anticancer activity of Ber has been demonstrated in various cancer types, including liver cancer [12], human gastric cancer [13], prostate cancer [14], endometrial cancer [15], pancreatic cancer [16], and ovarian cancer [17].

Berberine has shown the ability to inhibit cancer cell growth through different mechanisms, including apoptosis, cell cycle regulation, and autophagy modulation. To address its poor lipid and water solubility, the development of berberine derivatives became imperative [10,18]. Recently, the discipline of nanomedicine has made great progress in the development of new nanoparticles, particularly for cancer treatment [9].

Selenium (Se) is involved in the regulation of the immune response and in cancer prevention [19,20]. Selenium nanoparticles (SeNPs) are considered promising potential drug delivery systems and have shown a variety of benefits, including improved antitumor activity, reduced cytotoxicity, and high drug-loading capacity [21]. Thus, SeNPs have been used as an important anticancer medication delivery mechanism [22]. To harness the pharmacological benefits of selenium nanoparticles, it is crucial to consider factors such as the size of the particles, their chemical composition, and the amount administered [23].

Silver stands out as the most commercially viable precious metal for the fabrication of nanoparticles and nanomaterials. Its utilization is attributed to its ability to improve various physicochemical features compared to bulk materials, encompassing electrical, thermal, catalytic, and optical properties [24]. The advantages of silver nanoparticles in drug delivery systems include adjustable dimensions and configurations, dense attachment of surface ligands, the increased stability of nucleic acids bound to the surface, and enhanced precision in timed/controlled drug delivery within cells. Additionally, these nanoparticles offer protection for attached therapeutics from degradation [25]. AgNPs have been widely used in household utensils, healthcare, and diverse areas such as food preservation, environmental studies, and biomedical applications, due to their exceptional features [26]. These nanoparticles possess anti-neoplastic, antiangiogenic, antimicrobial, and anti-inflammatory properties that are highly beneficial for various purposes [27]. Interestingly, several studies demonstrated the biological and anticancer effects of silver nanoparticles [28,29].

Chemical, physical, and biological procedures are all used to produce nanoparticles. Green synthesis, which refers to the environmentally friendly and sustainable manufacturing of nanoparticles without the use of hazardous chemicals or toxic solvents, has gained popularity in biological processes in recent years [30]. Popular green synthesis processes use natural sources such as plants and microorganisms. This process has several advantages over traditional synthesis methods, including lower costs, greater scalability, less hazardous waste, and a high-performance structure that can be easily scaled up for industrial production. Furthermore, green synthesis can produce nanoparticles with distinct forms, sizes, and surface qualities that are customized for specific applications. Enzymes, proteins, polyphenols, flavonoids, and terpenoids, which can act as catalyzing, reducing, stabilizing, or capping agents for one-step synthesis, are among the biological sources used for the synthesis of green nanoparticle synthesis [31].

This work aimed to compare the in vitro antitumor activity of green synthesized berberine–silver nanoparticles (Ber-AgNPs) and berberine–selenium nanoparticles (Ber-SeNPs) in the human liver cancer cell line HepG2.

## 2. Materials and Methods

### 2.1. Chemicals and Reagents

Berberine (>99%), silver nitrates (AgNO_3_), sodium selenite (Na_2_SeO_3_), and dimethyl sulfoxide (DMSO) were purchased from Sigma-Aldrich Chemical Co. (St. Louis, MO, USA). Cisplatin (CDDP; Pt(NH_3_)_2_Cl_2_) (UNISTIN^®^, EIMC United Pharmaceuticals, Badr City, Cairo, Egypt) was used in this study as a positive control. Otherwise, all reagents utilized were of the highest purity.

### 2.2. Biosynthesis of Ber-AgNPs

The effective green synthesis of AgNPs followed the methodology outlined by El-Khadragy et al. [26]. Briefly, a 0.1 mM/mL aqueous solution of berberine was introduced into a solution containing 0.1 mM/mL AgNO_3_ and agitated at temperatures ranging from 45 to 50 °C. The size of the synthesized Ber-AgNPs, measured using a Zetasizer (ZEN 3600), averaged 215.4 ± 10.8 nm, with a mean zeta potential of −3.73 mV (Supplementary Data: Table S1 and Figure S1). The formed NPs had a Ber:Ag ratio equal to 3.1:1.

### 2.3. Biosynthesis of Ber-SeNPs

Combining two milliliters of berberine (0.1 mM/mL) with ten mL of Na_2_SeO_3_ (0.1 mM/mL) and stirring for a full day at room temperature resulted in the synthesis of Ber-SeNPs [10]. The average size of the Ber-SeNPs, measured using a Zetasizer (Nano series, ZEN 3600, Malvern, UK), was found to be 171.5 ± 4.2 nm, with a mean zeta potential of −12.4 mV (Supplementary Data: Table S1 and Figure S1). The formed NPs had a Ber:Se ratio equal to 4.3:1.

### 2.4. Cell Lines and Culture Conditions

The hepatocellular carcinoma cell line (HepG2) and the mouse normal liver cell line (BNL) were acquired from Nawah Scientific Inc., located in Mokatam, Cairo, Egypt. These cell lines were grown in T25 culture flasks, maintaining a density of 2 × 10^4^ cells, in Dulbecco’s modified Eagle medium (DMEM; Gibco, ThermoFisher Scientific, Waltham, MA, USA) supplemented with 10% fetal calf serum, 100 U/mL penicillin, and 100 IU/mL streptomycin. The culture was carried out in a humidified incubator with a 5% CO_2_ atmosphere at 37 °C, with media changes every 48 h. Passage occurred when cells reached 75% confluence under an inverted microscope. For cell collection, trypsinization with 0.025% trypsin and 0.02% EDTA was followed by washing in phosphate buffered saline (PBS).

### 2.5. Design of the Study

To assess the anticancer mechanism of the tested materials, HepG2 cells were divided into seven groups. One of them was incubated with Ber (13 µg/mL), and the other four groups were incubated with Ber-AgNPs and Ber-SeNPs (one-third or one-half of the IC_50_ of each group of NPs). Untreated cells were considered as a control; however, CDDP was used as a positive control (0.17 µg/mL). The design of the study is detailed in Table 1. Incubation was extended for 24 h.

### 2.6. Cytotoxicity Assay

The sulforhodamine B (SRB) assay was used to evaluate cell viability. In 96-well plates, 100 μL of HepG2 or BNL cell suspension (5 × 10^3^ cells) was incubated for 48 h in full medium. Another 100 μL of medium with varying drug concentrations was added for cell treatment with the vehicle, Ber, Ber-AgNPs, Ber-SeNPs, or CDDP. After 24 h of drug exposure, cells were fixed at 4 °C for one hour and the medium was substituted with 150 μL of 10% TCA. After removing TCA, cells were washed five times with distilled water. Subsequently, 70 μL of 0.4% *w*/*v* SRB solution was added and incubated for 10 min in a dark area. After three 1% acetic acid washes, plates were air-dried overnight. Subsequently, 150 μL of 10 mM TRIS was used to remove the SRB staining from proteins. The absorbance was measured at 540 nm using a microplate reader (Biotech, Inc., Minneapolis, MN, USA).

### 2.7. Wound Healing Cell Migration Assay

Cell migration was evaluated by investigating the cell capacity to migrate within the cellular environment in a 2D in vitro wound healing assay [32]. Briefly, HepG2 cells were seeded into six-well plates (2 × 10^5^ cells/well) one day before treatment with Ber, Ber-AgNPs, Ber-SeNPs, and CDDP. When the confluence reached ~90%, the cells were exposed to Ber, Ber-AgNPs, Ber-SeNPs, and CDDP for 24 h. Then, a horizontal scratch was performed, and the plates were washed with PBS to remove debris. Random fields were selected and photographed at 0, 24, 48, and 72 h. From this, the migrated distances were measured and the migratory abilities were detected as a ratio of the 72 h distance to the 0 h distance from the same field.

### 2.8. Lactate Dehydrogenase (LDH) Assay

To evaluate membrane integrity in the treated HepG2 cells and control, LDH activity was measured using the LDH kit (Abcam, Cambridge, UK). The leakage of LDH from the cells was determined in the medium and the quantification of the produced color was performed at 450 nm using a microplate reader (BioTek ELX800, Winooski, VT, USA). Cells at a density of 2 × 10^4^ cells were cultured with vehicle, Ber, or Ber-NPs for 24 h at the concentrations listed in Table 1. As a result, 10 µL of supernatant was incubated for 30 min with 100 µL of LDH mix (ab65393) and absorbance was measured at 450 nm.

### 2.9. Determination of Factors Related to Apoptosis and Inflammation

Abcam ELISA kits (Cambridge, UK) were used following the manufacturer’s guidelines for the colorimetric determination of mitochondrial apoptosis markers (Bcl-2, Bax, and cystolic cytochrome C), P53, and cleaved caspase-3. Furthermore, tumor necrosis factor alpha (TNF-α), cyclooxygenase (COX)-2, and nuclear factor kappa B (NF-κB) were determined as inflammatory markers. In brief, 2 × 10^6^ of HepG2 cells were cultured, and after confluence subjected to Ber, Ber-AgNPs, Ber-SeNPs, and CDDP at the concentrations tested and incubated for 24 h. After removing the medium by centrifuging the cells at 1800× *g* for 5 min, the cells were recovered and washed in PBS twice. A total of 50 microliters of cold lysing buffer was used to lyse the pellets. After centrifuging the resulting cell lysate for 1 min at 4 °C at 12,000× *g*, the supernatant was collected. The protein concentrations in each cell lysate were determined using the Bradford technique. When the protein content of the sample was more than 4 g/L, the cell extraction buffer PTR was used to dilute it. In the end, the color of the developed samples was assessed at 405 nm in a microplate reader (Biotech, Inc., USA).

### 2.10. Determination of Cell Cycle-Related Factors

The gene expression of cyclin D1 and cyclin-dependent kinase 2 (CDK2) confirmed the presence of cell cycle-related proteins in treated HepG2 and control cells. Following cell harvesting, total RNA was extracted using Trizol reagent (Life Technologies, Carlsbad, CA, USA) according to the manufacturer’s instructions. Quantification of isolated RNA was performed with nanodrop, and cDNA synthesis was conducted using RevertAid TM H Minus Reverse Transcriptase (ThermoFisher Scientific, Waltham, MA, USA). RT-PCR operations were performed using SYBR Green Supermix (Biorad, Hercules, CA, USA) on a ViiATM 7 system (Thermo Fisher Scientific, USA). The housekeeping control used was the β-actin gene. Detailed gene-specific primers for cyclin D1 and CDK2 can be found in Table 2. The results are presented as folds of change in gene expression compared to controls.

### 2.11. Determination of the Oxidative Status of Cells

Confluent HepG2 cells cultured in T25 cell culture flasks were treated for 24 h with Ber, Ber-AgNPs, Ber-SeNPs, and CDDP. After being harvested, cells were lysed in a lysing buffer and supernatant was collected after the lysate was centrifuged at 12,000× *g* for one minute at 4 °C. The supernatant was immediately used for the estimation of reactive oxygen species (ROS) using the green fluorescent strain 2,7-dichlorofluorescein diacetate (DCFH-DA) [9]. However, lipid peroxidation (LPO) and glutathione (GSH) were colorimetrically evaluated in the supernatant according to Ellman [33] and Ohkawa et al. [34], respectively. Total protein was estimated using the Bradford method in each cell lysate using bovine serum albumin as the standard [35].

### 2.12. Statistical Analysis

Results are presented as mean ± standard deviation (SD). To assess differences among groups, a one-way ANOVA was conducted, and multiple comparisons were performed using Tukey’s test. Statistical analysis was carried out using SPSS software (version 20.0). Significance was considered when the *p* values were smaller than 0.05.

## 3. Results

### 3.1. Cytotoxic Effect of Berberine and Its Nanoderivatives

**Figure 1:**
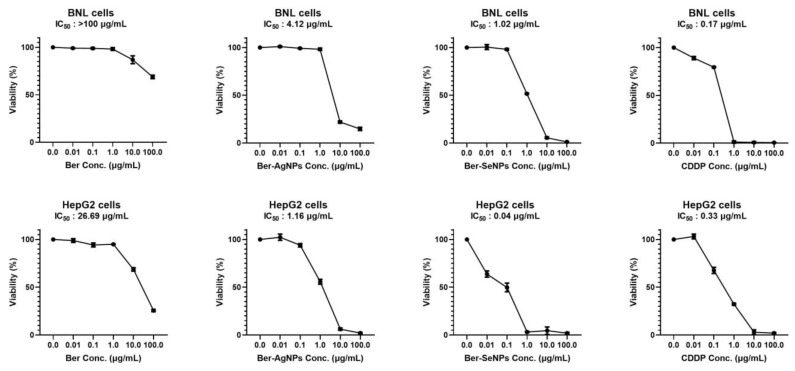
The viability of HepG2 and BNL cell lines after 48 h of incubation with berberine and its nanoderivatives (Ber-AgNPs and Ber-SeNPs) using the SRB assay. Incubation with serial concentrations (0.01–100 µg/mL) of the tested materials was carried out in triplicate. Data are presented as the mean ± SD of three separate experiments. Ber, berberine; CDDP, cisplatin.

The cytotoxic effect of berberine and its nano-derivatives Ber-AgNPs and Ber-SeNPs against HepG2 and BNL cells was investigated using the SRB assay (Figure 1). Taking into account IC50, the results revealed 26.69, 1.16, and 0.04 µg/mL for Ber, Ber-AgNPs, and Ber-SeNPs, respectively. However, cisplatin (CDDP) showed an IC_50_ of 0.33 µg/mL. Berberine nanoparticles significantly decreased the HepG2 cell IC50 compared to Ber alone by approximately 23 and 667 folds for Ber-AgNPs and Ber-SeNPs, respectively. However, normal BNL cells were less sensitive to the cytotoxicity exerted by the tested materials. IC_50_ was >100, 4.12, and 1.02 µg/mL for Ber, Ber-AgNPs, and Ber-SeNPs, respectively.

### 3.2. Berberine and Its Nanoderivatives Exhibit Antimigratory Properties against HepG2 Cells

**Figure 2:**
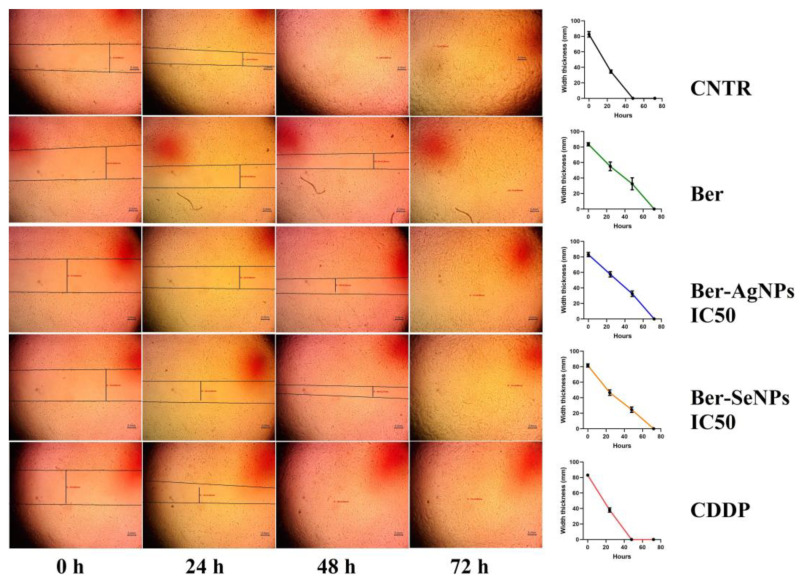
Migration capacity is determined by wound healing assay. HepG2 cells were treated with berberine and its nanoderivatives (Ag and Se), and the wound thickness exhibited a lower slope for closure comparable to control cells. After 48 h, the treated cells showed a clear gap, while the untreated HepG2 cells filled the bulk of the injured area. Vertical stripes indicate the width of the growth-free zone. Ber, berberine; CDDP, cisplatin.

To further test the anticancer properties of berberine and its nanoderivatives (Ag and Se), a wound-healing assay was carried out to investigate the effect of the materials tested on the migration of HepG2 cells (Figure 2). The results revealed that the rate of migration in the cells treated with berberine and its nanoforms (Ber-AgNPs and Ber-SeNPs) was significantly lower than in the control cells.

### 3.3. LDH Enzyme Leakage

**Figure 3:**
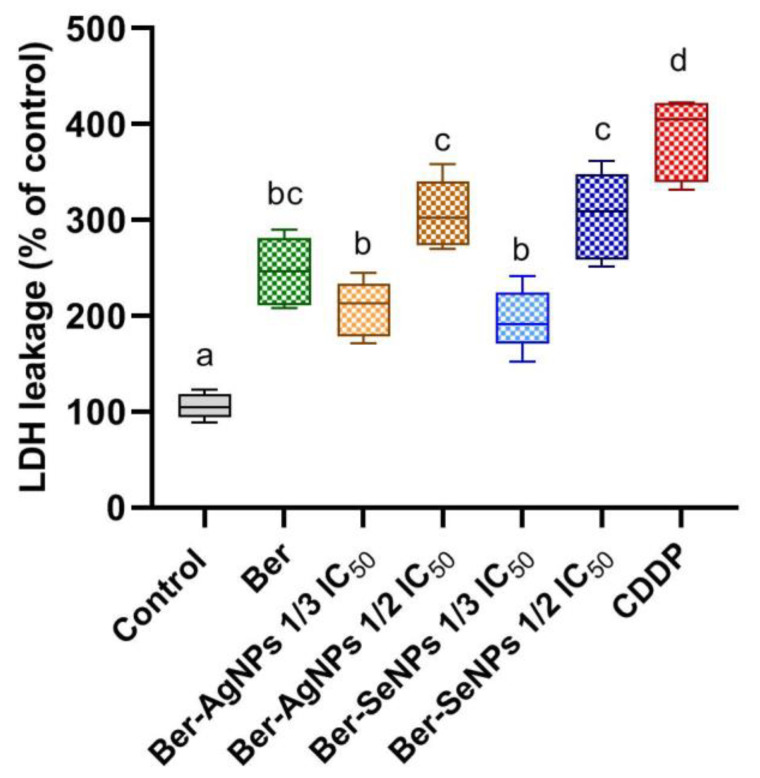
Lactate dehydrogenase (LDH) activity in HepG2 cells after 24 h of incubation with berberine and its nanoderivatives (Ber-AgNPs and Ber-SeNPs). Data were presented as (mean ± SD) from three separate experiments. Different letters indicate statistically significant differences at *p* < 0.05. Ber, berberine; CDDP, cisplatin.

The evaluation of cell membrane damage and loss of integrity was estimated using the LDH assay in treated and control HepG2 cells (Figure 3). Regarding untreated cells, incubation with Ber and both Ber-NPs (Ag and Se) led to a significant dose-dependent increase (*p* < 0.05) in LDH activities in the medium of the treated HepG2 cells. Treatment with one-third and one-half of the IC_50_ of Ber-AgNPs led to elevations of approximately 1.96 and 2.89 times, respectively, compared to the control. Similarly, treatment with one-third and one-half of IC_50_ of Ber-SeNPs led to increases of approximately 1.85 and 2.87 times, respectively, compared to the control.

### 3.4. Effect of Berberine and Its Nanoderivatives on HepG2 Cell Apoptosis

**Figure 4:**
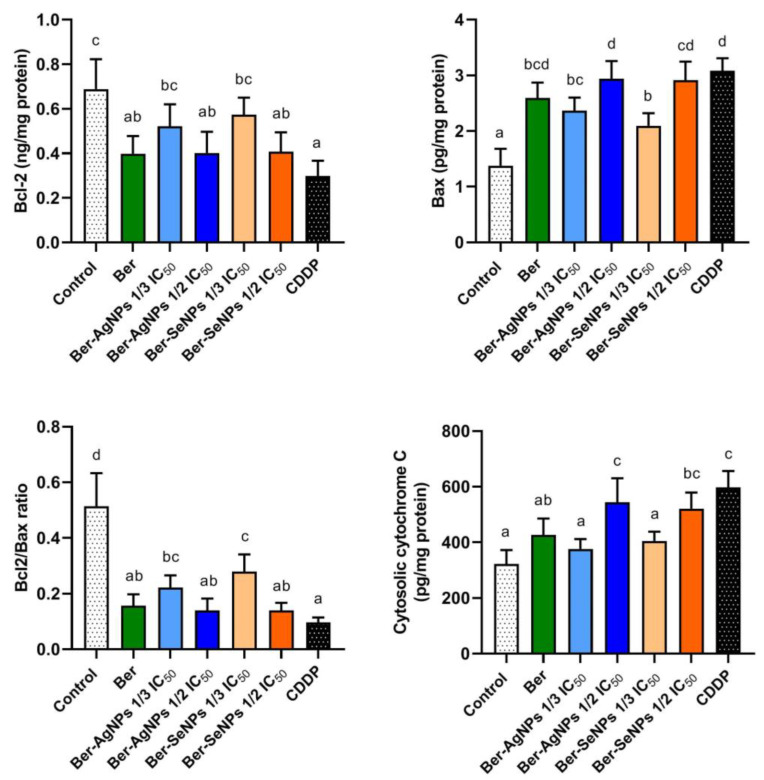
Changes in the levels of the regulatory apoptotic proteins Bcl-2, Bax, and cytochrome C in HepG2 cell lines after 24 h of treatment with berberine and its nanoderivatives. Data are presented as the mean ± SD from three separate experiments. Different letters indicate statistically significant differences at *p* < 0.05. Ber, berberine; CDDP, cisplatin.

To evaluate changes in mitochondrial apoptotic regulators after treatment with berberine and its nanoderivatives, levels of pro-apoptotic proteins (Bax and cytochrome C) and the anti-apoptotic protein (Bcl-2) were assessed. Furthermore, changes in the Bcl-2/Bax ratio were also evaluated (Figure 4). After incubation with one-third and one-half the IC_50_ of the Ber-AgNPs or Ber-SeNPs, a tendency of a decrease in Bcl-2 levels was observed, accompanied by an increase in Bax levels, compared to the control group. However, the cytochrome C showed a dose-dependent increase in both NP treatments. Furthermore, berberine caused obvious alterations in Bcl-2, Bax, and cytochrome C levels compared to the control and CDDP-treated cells. The Bcl-2/Bax ratio was significantly reduced in a dose-dependent manner by ~56.9 and 72.87% after treatment with one-third and one-half IC_50_ of the Ber-AgNPs, respectively, compared to the control cells. Similarly, treatment with one-third and one-half IC_50_ of Ber-SeNPs led to a significant decline in Bcl-2/Bax ratio by 45.79 and 72.8%, respectively, compared to the control. These results clarify the mechanisms of Ber and its nanoderivatives in inducing the intrinsic apoptotic pathway.

### 3.5. Changes in p53 and Caspase-3 Levels

**Figure 5:**
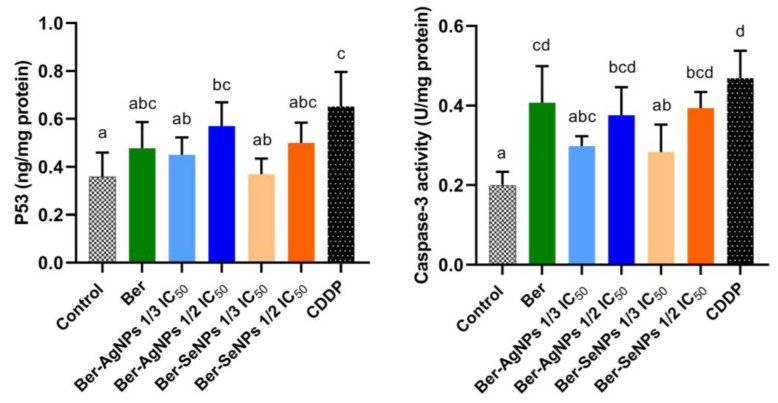
Alterations in p53 levels and caspase-3 activities in treated HepG2 cells and controls after 24 h. The effect of berberine and its nanoderivatives was investigated using an ELISA assay. Data are presented as the mean ± SD from three separate experiments. Different letters indicate statistically significant differences at *p* < 0.05. Ber, berberine; CDDP, cisplatin.

The alteration in the protein levels of p53 and caspase-3 was assessed (Figure 5). Regarding untreated cells, incubation with both Ber-NPs concentrations led to a nonsignificant increase in p53 levels in treated HepG2 cells, except for the higher dose of Ber-AgNPs, which showed a significant elevation by ~59.2%. Furthermore, caspase-3 activities showed a nonsignificant increase in the one-third IC_50_ of both Ber-NPs; however, a significant increase was observed in the higher concentrations (Ber-AgNPs and Ber-SeNPs) of ~89 and 97%, respectively, compared to the controls. These results suggest evidence of cell death via the p53-dependent apoptotic pathway, except for the lower dose of Ber-SeNPs, which exhibited p53-independent apoptosis.

### 3.6. Cell Cycle Regulators

**Figure 6:**
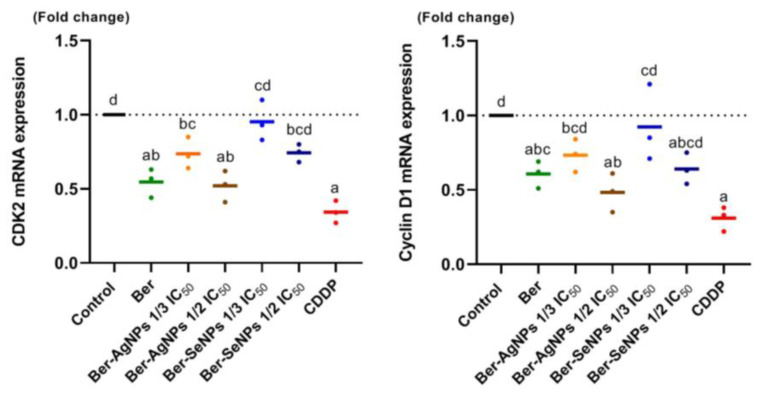
Changes in levels of the cyclin D1 and CDK-2 mRNA expression patterns in HepG2 cell lines after 24 h of treatment with berberine and its nanoderivatives. Data are presented as the mean ± SD of three separate experiments. Different letters indicate statistically significant differences at *p* < 0.05. Ber, berberine; CDDP, cisplatin.

CDK-2 and cyclin D1 expressions were assessed in treated and control HepG2 cells to evaluate the effects of Ber and Ber-NPs incubation (Figure 6). In the Ber-AgNPs groups, cyclin D1 and CDK 1 expression was significantly down-regulated (*p* < 0.05) in a dose-dependent pattern compared to the control cells at both concentrations (one-third one-half IC_50_). In the Ber-SeNPs groups, the higher dose (one-fourth IC_50_) showed a down-regulation of cyclin D1 and CDK-2 gene expression by approximately 0.4 and 0.25 times, respectively, compared to the controls. However, the lower dose of Ber-SeNPs (one-third IC_50_) did not show a considerable change. Maximum down-regulation was observed in the Ber-AgNPs (one-half IC_50_) groups (~52 and 48% for cyclin D1 and CDK-2, respectively). The decline in cyclin D1 expression suggests a cell cycle arrest in the G_1_ phase due to Ber-AgNPs incubation (both doses) and the higher doses of Ber-SeNPs. However, downregulation of CDK-2 contributed to G_2_/M arrest in all tested groups, except for the lower-dose Ber-SeNPs group.

### 3.7. Effect on the Inflammatory Mediators

**Figure 7:**
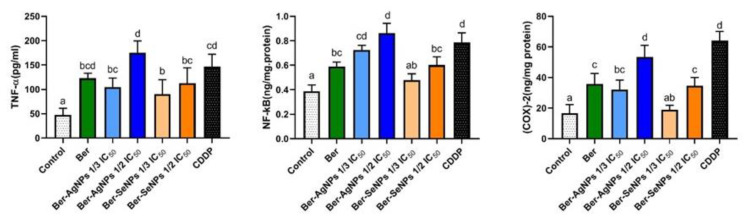
Changes in TNF-α, NF-κB, and COX-2 levels of HepG2 cells after 24 h of treatment with berberine and its nanoderivatives. Data are presented as the mean ± SD from three separate experiments. Different letters indicate statistically significant differences at *p* < 0.05. Ber, berberine; CDDP, cisplatin.

TNF-α, COX-2, and NF-κB were colorimetrically assessed in treated and control HepG2 cells (Figure 7). HepG2 cells incubated with berberine and its nanoderivatives exhibited a significant (*p* < 0.05) dose-dependent increase in TNF-α levels compared to control cells. The maximum value was observed in Ber-AgNPs (one-half IC_50_) with a significant increase compared to the Ber and control groups (1.4- and 3.69-fold, respectively). Similarly, incubation with Ber and both Ber-NP concentrations (Ag and Se) led to a significant (*p* < 0.05) dose-dependent elevation in NF-κB and COX-2 levels compared to the control cells. It should be noted that Ber-AgNPs achieved the maximum effect (one-half IC_50_) with a significant increase compared to the Ber and control groups.

### 3.8. Oxidative Status

**Figure 8:**
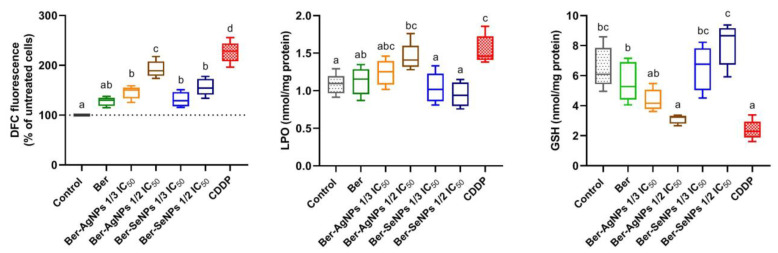
Oxidative stress on HepG2 cell lines after 24 h of treatments with berberine and its nanoderivatives. Data are presented as the mean ± SD from three separate experiments. Different letters indicate statistically significant differences at *p* < 0.05. Ber, berberine; CDDP, cisplatin.

The effect of berberine and its nanoderivatives on ROS, LPO, and GSH was evaluated in treated and control HepG2 cells (Figure 8). The results revealed that Ber and its NPs exerted oxidative stress on HepG2 cells. This effect was noticed with a significant dose-dependent increase in ROS after incubation with Ber and Ber-NPs with respect to control cells. Furthermore, Ber-AgNPs exhibited oxidative stress in both treated groups by increasing the LPO, with a significant increase of ~33.74% in the higher one; however, Ber-SeNPs showed a nonsignificant decrease. Ber-AgNPs treatments caused a dose-dependent decline in GSH levels with significant (*p* < 0.05) records in the higher one (one-half IC_50_), which decreased by ~53% comparable to the control cells. However, the Ber-SeNPs dose-dependently elevated GSH levels in both treated groups with nonsignificant records with respect to the control cells.

## 4. Discussion

Berberine is a natural herbal alkaloid found in the roots and barks of *Berberis* sp. and has remarkable biological and pharmacological activities as an anti-inflammatory, antidiabetic, antimicrobial, anti-oxidant, and anticancer agent [10,36]. In this research, we developed berberine nanoparticles based on silver and selenium in a trial to enhance berberine absorption and bioavailability in target cells. The anticancer properties of berberine were extensively reported [10,36,37,38]. Our results reveal that berberine and its Ag and Se nanoderivatives exerted cytotoxic and antimigratory potencies in vitro against HepG2 cells. Furthermore, the in vitro cytotoxic effect of Ber-AgNPs against MDA-MB-231 and MCF-7 cancer cell lines was proven by Bhanumathi et al. [39]. Moreover, the anticancer potency of Ber-SeNPs was previously reported in vivo as reducing tumor size and cell proliferation in Ehrlich solid tumors [10]. Our results are in accordance with those of Wang and Zhang [15], who reported that Ber suppresses the in vivo and in vitro migration of endometrial cancer cells by inhibiting the miR-101/COX-2/PGE2 signaling cascade. Additionally, Liu et al. [40] showed that Ber decreased the viability and inhibited the migration of Panc-1 and hTERT-HPNE pancreatic cancer cells by regulating the citrate metabolism and transport in mitochondria. In cytotoxic potency, Ag and Se berberine nano-derivatives showed a greater effect than berberine alone, and this is in agreement with Sahibzada et al. [41], who suggested that berberine nanoparticles had better biological activity than unprocessed Ber, which may be ascribed to enhanced solubility and bioavailability. Furthermore, Ber-SeNPs were superior to Ber and Ber-AgNPs, which may be attributed to their original anticancer properties [42,43].

The magnitude of the zeta potential indicates the possible stability of the Ber-SeNPs. If the zeta potential increases, there will be an increased repulsion between particles, leading to a more stable dispersion of particles. If all suspended particles have a strong zeta potential that is negative or positive, they seem to repel each other, and the particles are not likely to join together [44]. In the end, this helps to better penetrate and deliver Ber-SeNPs particles.

Lactate dehydrogenase is a stable cytoplasmic enzyme found in almost all living cells; its release outside the cell is a crucial feature of cell damage [45]. In this study, we quantified LDH in cell culture media as a remarkable sign of cytotoxicity. Our results reveal that Ber and both Ber-NPs (Ag and Se) led to a significant dose-dependent increase in LDH activities in the medium of treated HepG2 cells, which confirms the cytotoxic potentials of Ber and its nanoderivatives. These results are in the same line as Wang et al. [46], who clarified that Ber stimulated the release of LDH from IMCE and HT-29 colon cancer cell lines in a manner dependent on concentration. Similarly, Ber was approved to increase LDH release in the triple-negative breast cancer MDA cell line of triple-negative breast cancer [47]. Our results show that one-half IC_50_ of both Ber-AgNPs and Ber-SeNPs exhibited more LDH release in culture media than Ber. This is in accordance with previous literature that proved that Ber nanomaterials, such as berberine-loaded silver nanoparticle bioformulation of silver nanoparticles [39], berberine-loaded disulfide-bridged mesoporous organosilica nanoparticles [48], nanoparticles loaded with berberine [49], and SeNPs [10], boost berberine’s cytotoxic potency against different cancer cells, and due to their nanosize, they become easier for cells to absorb.

In this paper, the results show that Ber and its Ag and Se nanoparticles exerted a good antitumor effect against HepG2 cells by inducing apoptosis by up-regulating Bax, cytosolic cytochrome C levels, and caspase-3 activity, along with down-regulating Bcl-2 levels. HepG2 cells express wild-type functional p53 that showed up-regulation after the treatments in this study. This up-regulation in addition to those above suggests evidence of cell death via the p53-dependent apoptotic pathway, except for the lower dose of Ber-SeNPs, which exhibited p53-independent apoptosis. Furthermore, it leads to G_1_ cell cycle arrest by depleting the expression of cyclin D1 and CDK-2 mRNA. Similarly, earlier research has shown that berberine hinders lung cancer cell growth by affecting the matrix metalloproteinase 2 (MMP-2)/Bcl-2/Bax and Janus kinase 2 (Jak2)/vascular endothelial growth factor (VEGF)/NF-κB/AP-1 signaling pathways [50]. Additionally, it induces arrest of the G1 cell cycle through the Akt/CREB signaling axis [51]. Additionally, berberine was found to trigger apoptosis by activating the intrinsic pathway, as it activates caspase-3 and caspase-8, which in turn release cytochrome C, in addition to promoting ROS production in tumor cells [50,52]. Furthermore, previous work confirmed that berberine induced cell cycle arrest by upregulating levels of p21, p27, and p38 together with downregulating levels of cyclin A, cyclin D, CDK1, and CDK4 [53,54]. Furthermore, berberine-derived silver nanoparticles induce apoptosis in breast cancer cells by suppressing the expression of HIF-1α through the inhibition of the expression of the PI3K/AKT and Ras/Ras/ERK protein in signaling pathways and the generation of reactive oxygen species (ROS) [55]. Consistent with this, Othman et al. [10] showed that Ber-SeNPs caused apoptosis by increasing Bax levels and caspase-3 activity while decreasing Bcl-2 levels in a model of Ehrlich solid tumors in mice.

HepG2 cells express mRNAs for several cytokines and cellular regulators, including tumor necrosis factor (TNF)-α [56]. Our results demonstrate that berberine and its applied nanoderivatives promoted dose-dependent elevations in TNF-α, NF-κB, and COX-2 levels in HepG2 cells; notably, the highest level of these parameters was obtained after treatment with one-half IC_50_ of Ber-AgNPs. Consequently, the increased levels of these inflammatory mediators in the supernatant of HepG2 cells demonstrated that berberine and its applied nano-derivatives can activate the apoptotic pathway by upregulating inflammatory factors [57,58]. Moreover, Wang et al. [59] reported the ability of berberine to up-regulate TNF-α, which in turn enhances apoptosis in the hepatocellular carcinoma cell line (SMMC-7721).

An effective treatment strategy is to induce oxidative stress, since cancer cells are very vulnerable to ROS. Our results indicate that Ber and its NPs caused oxidative stress in HepG2 cells. This influence was shown with a significant dose-dependent increase in ROS and LPO and a decrease in GSH levels after incubation with Ber and Ber-NPs. Nanoparticles have the ability to infiltrate cells, generate ROS, and inhibit antioxidant molecules as a result of their diminutive dimensions and expansive surface area. Ber enhances the rate of ROS generation in tumor tissues, thus inducing oxidative stress. Berberine enhances the activity of oxidative stress of SeNPs and AgNPs, leading to the activation of apoptosis and inflammatory pathways. These findings are consistent with earlier research [50,52]. However, several studies have shown that berberine exhibits anti-inflammatory effects [60,61]. Therefore, the effect of induced inflammation on normal cells still awaits further investigation, particularly in an appropriate in vivo model.

This study has some limitations; although the results of the current study demonstrate that the biosynthesized Ber-NP derivatives are promising for potential future applications in the development of new anticancer drugs, more molecular, pharmacologic, and toxicologic experiments in vivo and in vitro are needed to confirm the safety and efficacy of these compounds, to gain more insight into the precise underlying mechanism of action, and to determine the optimal dose and route of administration.

## 5. Conclusions

We conclude that Ber and both Ber-NPs (Ag and Se) can inhibit the growth and migration of the hepatocellular carcinoma cell line (HepG2) by triggering oxidative stress and apoptotic cascades. The anticancer activity of Ber nanoparticles exceeds that of unprocessed Ber, perhaps because of their improved solubility and bioavailability. Furthermore, Ber-NPs exhibited greater efficacy compared to Ber, potentially due to the inherent anticancer characteristics of selenium and silver. More in vivo investigations are still needed to confirm our results.

## Figures and Tables

**Table 1:** Study design of HepG2 cells exposed to different treatments.

Group	Treatment Dose	Exposure Time
Group 1: Control	Vehicle	24 h
Group 2: Ber	13 µg/mL	
Group 3: Ber-AgNPs 1/3 IC_50_	0.4 µg/mL	
Group 4: Ber-AgNPs 1/2 IC_50_	0.6 µg/mL	
Group 5: Ber-SeNPs 1/3 IC_50_	0.013 µg/mL	
Group 6: Ber-SeNPs 1/2 IC_50_	0.02 µg/mL	
Group 7: CDDP	0.17 µg/mL	

**Table 2:** Sequences of the qPCR primers.

Gene	Accession Number	Forward (5′–3′)	Reverse (5′–3′)
Cyclin D1	NM_053056.3	GAGGCGGAGGAGAACAAACA	GGAGGGCGGATTGGAAATGA
CDK2	NM_001290230.2	GACACGCTGCTGGATGTCA	GAGGGGAAGAGGAATGCCAG
β-actin	NM_001101.5	AGCCTCGCCTTTGCCG	CGCGGCGATATCATCATCCA

## Data Availability

All relevant data are given within the paper.

## Supplementary Materials

The following supporting information can be downloaded at: https://www.mdpi.com/article/10.3390/cells13030287/s1. Table S1 and Figure S1: The characterization of berberine-loaded silver nanoparticles (Ber-AgNPs) and berberine-loaded selenium nanoparticles (Ber-SeNPs); the hydrodynamic diameter was determined using a Zetasizer and the surface charge was determined by calculating the Zeta potential.
