# Supplementary material for: Green-Synthesized Silver and Selenium Nanoparticles Using Berberine: A Comparative Assessment of In Vitro Anticancer Potential on Human Hepatocellular Carcinoma Cell Line (HepG2)
Source: Cells. 2024 Feb 5;13(3):287. doi: 10.3390/cells13030287 (PMC10854975; doi:10.3390/cells13030287)
Supplement: Supplementary file 1 [file cells-13-00287-s001.zip › cells-2821316-supplementary.pdf]

Supplementary data; Table S1 and Figure S1: Characterization of berberine loaded silver nanoparticles (Ber-AgNPs) and berberine loaded selenium nanoparticles (Ber-SeNPs) determined hydrodynamic diameter by Zetasizer and surface charge by Zeta potential.

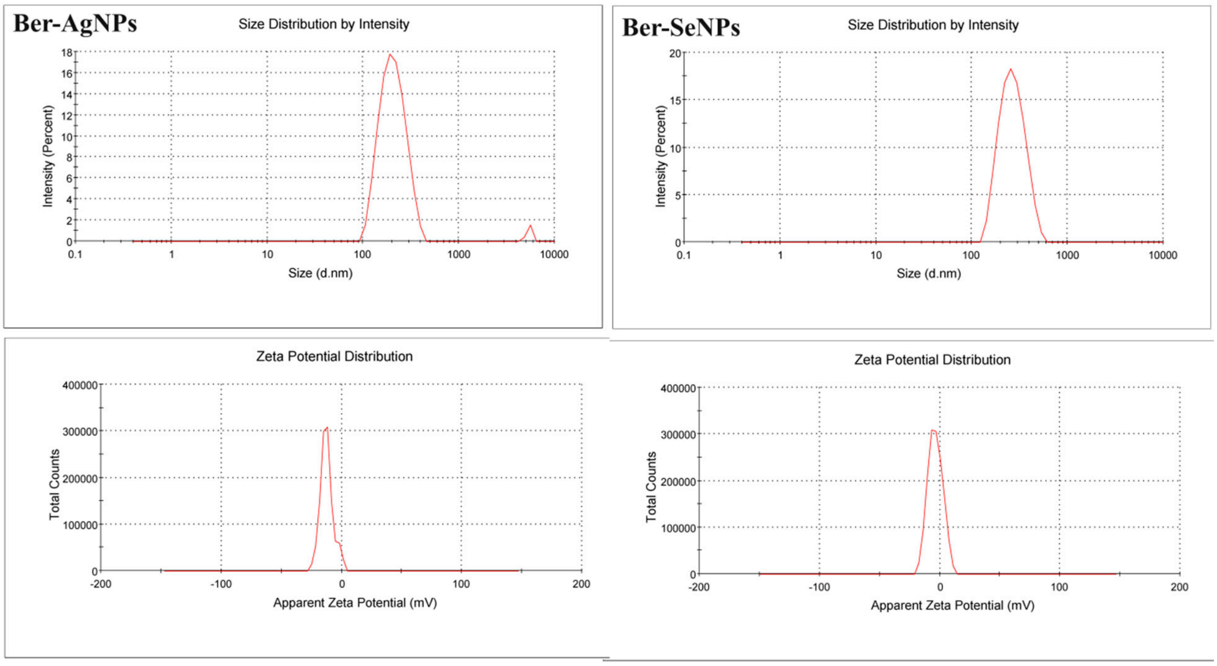

NPs	Hydrodynamic diameter (nm)	Surface charge (mV)
**Ber-AgNPs**	215.4±10.8	-3.73
**Ber-SeNPs**	171.5±4.2	-12.4
